# Leveraging Responsible, Explainable, and Local Artificial Intelligence Solutions for Clinical Public Health in the Global South

**DOI:** 10.3390/healthcare11040457

**Published:** 2023-02-04

**Authors:** Jude Dzevela Kong, Ugochukwu Ejike Akpudo, Jake Okechukwu Effoduh, Nicola Luigi Bragazzi

**Affiliations:** 1 Laboratory for Industrial and Applied Mathematics (LIAM), Department of Mathematics and Statistics, York University, Toronto, ON M3J 1P3, Canada; 2 Africa-Canada Artificial Intelligence and Data Innovation Consortium (ACADIC), York University, Toronto, ON M3J 1P3, Canada; 3 Global South Artificial Intelligence for Pandemic and Epidemic Preparedness and Response Network (AI4PEP), York University, Toronto, ON M3J 1P3, Canada

**Keywords:** artificial intelligence, big data and big data analytics, capacity development, digital public health goods, research and development, health data, research infrastructure, sustainable development, transdisciplinarity

## Abstract

In the present paper, we will explore how artificial intelligence (AI) and big data analytics (BDA) can help address clinical public and global health needs in the Global South, leveraging and capitalizing on our experience with the “Africa-Canada Artificial Intelligence and Data Innovation Consortium” (ACADIC) Project in the Global South, and focusing on the ethical and regulatory challenges we had to face. “Clinical public health” can be defined as an interdisciplinary field, at the intersection of clinical medicine and public health, whilst “clinical global health” is the practice of clinical public health with a special focus on health issue management in resource-limited settings and contexts, including the Global South. As such, clinical public and global health represent vital approaches, instrumental in (i) applying a community/population perspective to clinical practice as well as a clinical lens to community/population health, (ii) identifying health needs both at the individual and community/population levels, (iii) systematically addressing the determinants of health, including the social and structural ones, (iv) reaching the goals of population’s health and well-being, especially of socially vulnerable, underserved communities, (v) better coordinating and integrating the delivery of healthcare provisions, (vi) strengthening health promotion, health protection, and health equity, and (vii) closing gender inequality and other (ethnic and socio-economic) disparities and gaps. Clinical public and global health are called to respond to the more pressing healthcare needs and challenges of our contemporary society, for which AI and BDA can help unlock new options and perspectives. In the aftermath of the still ongoing COVID-19 pandemic, the future trend of AI and BDA in the healthcare field will be devoted to building a more healthy, resilient society, able to face several challenges arising from globally networked hyper-risks, including ageing, multimorbidity, chronic disease accumulation, and climate change.

## 1. Introduction

In the present paper, we will explore how new technological and digital tools can help address clinical public and global health needs in the Global South [1,2], leveraging and capitalizing on our experience with the “Africa-Canada Artificial Intelligence and Data Innovation Consortium” (ACADIC) Project in the Global South, and focusing on the ethical and regulatory challenges we had to face.

ACADIC is a newly formed interdisciplinary consortium that brings together a unique multidisciplinary team consisting of clinical public and global health experts, physicists, software engineers, data scientists, biostatisticians, epidemiologists, mathematicians, and biomathematical modelers, from 19 research centers and institutions/organizations, based in several African countries, such as South Africa, Nigeria, Cameroon, Rwanda, Namibia, Botswana, Zimbabwe, Mozambique, and Eswatini, as well as from Canada. 

The present “communication” paper was designed as a narrative literature review followed by a qualitative report of our experience with the ACADIC Project in the Global South, aimed at overviewing the main challenges of contemporary medicine with a “Global South” lens to inform the execution of future initiatives in the Global South.

## 2. Material and Methods

Concerning the first part (narrative literature review), we searched PubMed/MEDLINE, a major electronic scholarly database, which represents the most important and commonly utilized biomedical repository. We looked for the following keywords: “Global South”, “public health”, “global health”, “clinical public health”, “clinical global health”, “artificial intelligence”, “big data”, “big data analytics”, “Internet of Things”, “disruptive technologies”, “innovative technologies”, “digital tools”, “ethical issues”, “ethical challenges”, “regulatory issues”, and “regulatory challenges”.

Concerning the second part (qualitative report), we carried out a thematic analysis (TA) [3] of the reports submitted by the countries’ members of the ACADIC project. TA is one of the most commonly used approaches in the field of qualitative research, resulting in the discovery of “themes” (i.e., meaningful patterns consisting of implicitly/explicitly articulated ideas). The identification and emergence of these themes are enabled by the development of proper codes, that are iteratively revised and refined until “saturation” has been reached—that is to say, no further themes can be found and the identified themes extensively cover and entirely map all the texts under consideration. 

To ensure the procedure is adequately carried out, the team initially familiarizes itself with the reports, then, the texts are re-read and coded, subsequently re-examined, re-assessed, and, if necessary, codes are reviewed, and re-analyzed. The coding procedure is assisted by frequency and co-occurrence analyses and the formulation of research questions. New themes can emerge and be identified: if these are considered thematic variations of already identified themes, they are merged and combined, otherwise, they are added to the list of themes. 

All qualitative analyses were conducted using ATLAS.ti qualitative analysis software (version 23; ATLAS.ti GmbH, Berlin, Germany). 

## 3. Results 

In the following sections, we will briefly overview the main challenges of contemporary medicine with a “Global South” lens.

### 3.1. Clinical Public and Global Health 

Despite the lack of consensus on their precise definitions, health and well-being are not seen anymore as just the absence of disease, illness, or impairment/infirmity/disability, in a narrowly focused way, strictly entailing physical aspects from a mere biomedical context. Health and well-being are, rather, considered as the dynamic outcomes of a complex interplay of several parameters, which encompass various domains, including physical, psychological (mental and emotional), and social factors, among others [4,5,6]. 

Health and well-being can be investigated from various standpoints and scales, from the individual to the community/population perspectives, along what is known as the “healthcare continuum”. This includes disciplines such as clinical medicine, public and global health, each one of which consists of four major components: namely, (i) prevention by means of disease and disease outbreak monitoring/surveillance and early warning systems (EWSs)—that is to say, the effective collection, integration, interpretation, and deployment of data to enhance and support data-driven monitoring and surveillance of diseases and disease outbreaks, with a focus on systems that can help strengthen preparedness and early response, (ii) identification by means of clinical, public, and global health laboratory innovation—that is to say, an integrated laboratory and facility network, which is equipped with state-of-art infrastructure to provide all primary and specialist diagnostic services needed for disease and disease outbreak care, treatment, and prevention, (iii) individual and community/population health risk management by means of an integrated framework for disease and disease outbreak risk reduction and mitigation, and, finally, (iv) evidence-informed, data-driven decision-making processes [7,8,9,10] that shape and guide all previously mentioned, inter-related domains, from disease and disease outbreak prevention to identification, treatment, and recovery, both at the individual and community/population levels (Table 1).

Within the healthcare continuum, given the interrelatedness of the different domains, new specialties are arising. “Clinical public health” can be defined as an emerging, cutting-edge, multi-, cross-, and inter-disciplinary field [11], at the intersection of clinical medicine and public health, characterized by multi- and inter-sectoral collaboration, new disciplines, sub-specialties, and insights [12], as well as innovation, and strategic, forward-thinking [13]. Rather than focusing on the classical differentiation and separation between preventative and clinical approaches, as carried out by the prominent epidemiologist Geoffrey Rose [14,15], clinical public health combines both strategies, integrating primary care, clinical practice, disease management, treatment, and prevention, along the healthcare continuum [16]. Clinical public health is not just the practice of individual medicine (i.e., clinical medicine) on a larger scale, pursued by treating and managing cohorts of individuals (community/population) [14,15], but, rather, the acknowledgment of the interconnectedness of individual and community/population health. The former is, indeed, increasingly influenced and shaped by a wide array of community and population determinants of health. It is not possible to improve and enhance individual health, without drawing on principles of community/population and public health (and vice versa), combining leadership, clinical medical, and preventative care, and without advocating for stronger, more just health policies and health systems [11].

Furthermore, in the last decades, an innovative discipline called “clinical global health” [17,18] has emerged, which is the practice of clinical public health with a special focus on health issue management in resource-limited settings and contexts [19,20,21,22], which makes the acknowledgment of societal determinants of health more compelling and relevant, by incorporating the principles of equity, diversion, and inclusion (EDI).

As such, clinical public and global health represent vital approaches, absolutely instrumental in (i) applying a community/population perspective to clinical practice as well as a clinical lens to community/population health [8], (ii) identifying health needs both at the individual and community/population levels, (iii) systematically addressing the determinants of health, including the social and structural ones, (iv) reaching the goals of population’s health and well-being, especially of socially vulnerable, underserved communities, (v) better coordinating and integrating the delivery of healthcare provisions, (vi) strengthening health promotion, health protection, and health equity, according to EDI principles, and (vii) closing gender inequality and other (ethnic and socio-economic) disparities and gaps [23,24].

Clinical public and global health are called to respond to the more pressing healthcare needs and challenges of our contemporary society [25] (Table 2). 

### 3.2. Healthcare Needs and Challenges

In the last decades, health systems worldwide have been facing different, unprecedented challenges, such as the increase in the burden of disease (either communicable or non-communicable), especially in developing countries [26], mostly driven by an array of compounding, interacting, and interconnected factors. These include socio-demographic variables, aging and epidemiological transitions, antimicrobial resistance, climate change, urbanization, globalization, and accelerating human encroachment into natural landscapes such as forests, among others [27]. All this may lead to a greater demand for and utilization of healthcare services and provisions, and rising health costs, thus putting further stress on health systems, cascading across socioeconomic boundaries, disproportionately impacting vulnerable and marginalized populations, and magnifying societal disparities and gaps [28]. 

The novel Coronavirus (COVID-19) pandemic is just the latest in a series of global disease outbreaks caused by emerging (or re-emerging) infectious diseases (ERIDs), including the H1N1 influenza virus pandemic, the Severe Acute Respiratory Syndrome (SARS), and the West African Ebola epidemics [29], among others, which have posed an increasing threat to the individual well-being, as well as to the public and global health security and significantly put pressure and strain on already weak health systems. All this has widened underlying inequities and disparities and has stressed the urgent need of building and developing more strengthened, resilient, and rapidly responsive infrastructure and facilities [30]. 

These outbreaks and, in particular, the still ongoing COVID-19 pandemic have significantly challenged (i) the capacity and ability of healthcare workers to absorb unforeseen health system shocks and external events [31], (ii) to deliver high-quality healthcare provisions to all those who require them, and (iii) to preserve and maintain systems functioning and patient-centeredness [30].

Health policies responded with a profound restructuring and reorganization of health systems and medical services to better protect healthcare personnel itself and patients [32]. However, this restructuring was not enough, in many cases, to protect vulnerable, at-risk populations [33,34]. This warrants an innovatively framed health policy and systems research agenda to strengthen health systems in the face of pandemics/epidemics and other health system shocks and make them become more effective and quickly adaptative, as well as socially just, inclusive, equitable, and people-centered [30], conjugating precision, fairness, and inclusion [35,36] and “illuminating social position as a fundamental determinant of health and health inequities” [35]. 

Traditional approaches that inform risk assessment and management strategies as well as risk reduction and mitigation efforts aimed at addressing the complex (epidemiological, clinical, societal, and economic-financial) effects of diseases, disease outbreaks, and other public and global public health emergencies assume approximately linear relationships that link from a well-defined source to a single endpoint, and heavily rely on historical data, time series, and observations. Moreover, they are merely reactive, rather than proactive [37], and focus on consequences and impacts within a system that is spatially and temporally self-contained [38]. 

These approaches have proven to be inadequate for dealing with the challenges presented by the systemic nature of risk and vulnerability, namely, the “multifaceted interconnectedness of disease outbreaks, poorly understood breadth of population exposure, and profound nuance and detail of vulnerability” [39]. These deficits are starkly illustrated by the still ongoing COVID-19 pandemic, including the lack of an operationally defined, and testable theoretical and governance framework for systemic risk analysis, as well as inadequate operational practices for disease and disease outbreak risk mitigation and response [39].

As a consequence, the global community has failed to sufficiently and proactively identify, and respond to secondary impacts that intensify as “globally networked hyper-risks” [40] generated by “strongly connected, global networks” and complex, interacting, “highly interdependent systems”, cascading across various sectors and scales—local, regional, national and international, individual, community/population, public, and global [39,40,41]. In addition, existing risk management and governance infrastructure for disease and disease outbreak policies often lack adequate mechanisms for considering the diverse needs of vulnerable or at-risk populations, including those living in informal settlements and geographically isolated settings [42], socio-economically deprived or underserved populations [43,44], those who are homeless [45], racialized visible minorities [46,47], women [48], the elderly [49], persons with disabilities [50], Indigenous communities [51], informal workers [52], migrants and refugees [53], those without citizenship rights [54,55], sex workers [56,57,58], and the two-spirit, lesbian, gay, bisexual, transgender/transsexual, queer, intersex, asexual, polysexual/pansexual (2SLGBTQIAP+) community [59], among others. 

Learning and adaptation in the short-term response to a disease or an epidemic/pandemic can be enhanced by identifying the capacities and integrating the acute needs of the most vulnerable. Moreover, long-term transformation must incorporate new approaches to disease, pandemic and epidemic prevention, preparedness, management, response, and recovery, to address the systemic nature of risks [60]. Some studies [61,62] have hypothesized six potential pathways explaining why diseases (including multimorbidity and disability development and chronic disease accumulation) and disease outbreaks disproportionately affect minorities and vulnerable communities. These pathways include differential exposures and vulnerability to the pathogen/disease with differential health and societal consequences of the infection/disease, as well as differential effectiveness and adverse consequences of disease or epidemic/pandemic control measures [61]. Of note, disease or epidemic/pandemic control measures could have differential effectiveness and differential adverse consequence profiles, compared to the general population [63]. However, most of these pathways are poorly understood, warranting further studies. 

Moreover, diseases and disease outbreaks, especially COVID-19, have underlined the need for timely, accurate, and reliable data, to better inform public health decision-making in an evidence-based fashion. As such, data science has played a key role in the response to the still ongoing COVID-19 pandemic, driving measures and shaping interventions to mitigate its burden [64]. An incredible wealth of data from diverse, heterogeneous sources has been made available: ranging from human mobility, to contact tracing, medical imaging, basic and translational sciences, including virology and infectious diseases, chemistry, pharmacology, and drug screening and discovery, as well as bioinformatics, scholarly literature, and different parts of the health system—including the formal health care system and other systems that form part of the intersectoral action needed to strengthen health systems (e.g., housing, transportation, education—and also across public and private sectors). However, quoting the prominent philosopher Immanuel Kant, data without concepts (models and frameworks) are blind, as well as concepts (models) without content (data) are empty. 

Basically, a “fundamental redesign” and a “knowledge and paradigm shift in thinking” [40] with new conceptualizations, adaptive models, and operational tools—that incorporate the systemic nature of risks in an objective, comprehensive manner, and focus on the most vulnerable, extending “past individual patients to entire populations and geographies … across lifespan” [37]—are required to transform existing epidemic/pandemic response approaches which have demonstrated many weaknesses, given the sustained threat of ERIDs and other “globally networked hyper-risks” [40], ideally a “global systems science” approach [40], such as the “One Health” framework [65,66,67,68]. “One Health” [65] and “planetary health”/“clinical planetary health” [66,67] imply strategies that recognize the health of humans, domestic and wild animals, plants, and the wider environment/the entire ecosystem are profoundly linked, interconnected, and interdependent [68].

By definition, “One Health” and “planetary health”/“clinical planetary health” approaches require a system science approach (the so-called “one science”) [40,65,69] and an extensive toolbox, being characterized by a strong emphasis on the so-called 4 Cs [69] (namely, (i) capacity building, (ii) collaboration, (iii) coordination, and (iv) communication), is urgently needed. All this should enable the development and use of inter- and transdisciplinary models, to inform disease and disease outbreak prevention, surveillance, and response at the human-animal-ecosystem interface. 

Since ERID-related challenges, as well as other challenges, including economic-financial, geopolitical, environmental, technological, societal, and health “network hyper-risks” [40], are global in scale, international communication and shared, orchestrated strategies, building and capitalizing on the expertise developed in varied settings and contexts, as well as in different experiences and circumstances, are warranted to successfully address them. Artificial Intelligence (AI) ad Big Data Analytics (BDA) can help address these challenges. 

### 3.3. Artificial Intelligence and Big Data Analytics 

AI and BDA techniques have developed rapidly over the last 10 years [70]. New technologies and policies allowing the generation, collection, storage, handling, pre–processing, and processing of large, dynamical datasets continuously under evolution have enabled novel methods of analysis and implementation. Contemporary advancements and achievements in the field of data science and, in particular, BDA enable information visualization and predictions on an unprecedented scale, while AI-based technologies allow new information to be automatically analyzed by dynamic models, and provide accurate, precise, and reliable forecasts based on these analyses.

The advances in AI [71], including machine learning, and, in particular, deep learning (such as generative adversarial networks or GANs), as well as in disruptive technologies (such as the Internet of Things, IoT), have enabled the expansion of the potential scope of classical systems adopted in clinical medicine (from diagnosis to prognosis and management recommendations, patient engagement and compliance with treatment, and administrative activities, among other healthcare tasks), as well as traditional disease and disease outbreak (epidemic and pandemic) prevention, preparedness, and response mechanisms by making new data sources available to be integrated into multi-level analyses. Such changes provide unique opportunities to address gaps in existing surveillance systems, such as increasing their sensitivity and specificity for early detection of ERIDs and strengthening capabilities/capacity to enhance early warning, early response, and mitigation and control of developing disease and disease outbreak (epidemics/pandemics) [72]. 

In the last years, scholars have discussed the hypes and hopes of AI- and BDA-based tools and techniques at the individual and community/population levels, in the arena of clinical public and global health, where they can serve as tools to assist public and global health policies and decision-making processes in an informed, evidence-based, data-driven fashion [71]. Conversions and applications of these tools to public and global health practice have been occurring for the past few years, creating a baseline for future advancements and enabling us to overcome existing shortcomings. 

As stated by Scoones et al. [73], currently available and commonly utilized models look at diseases and disease outbreaks and the risks they pose and generate from single perspectives, rather than being integrative. As such, there is an urgent need for next-generation 3P (process-, pattern-based, and participatory) modeling that can capture disease and disease outbreak principles and functions, incorporate statistical associations and correlations between variables of interest, from an “ecological” standpoint, enriching the previous purely mechanistic perspective, and can combine the expertise of the modelers with the true needs and expectations of local communities, making the model locally relevant, informed, and contextualized [74].

### 3.4. Artificial Intelligence and Big Data Analytics in Clinical Public and Global Health 

AI and BDA can help face healthcare needs and challenges, being the catalyst for a profound transformation in the healthcare arena [71] and helping improve efficiency, effectiveness, and responsiveness, as well as equity in the delivery of public health and healthcare services [75], by developing and facilitating current practices, and by introducing new methods of surveillance and action both at the individual (clinical medicine) and community/population (public and global health) levels. 

As such, AI and BDA can affect all four facets of clinical medicine, and public and global health, profoundly reshaping them. AI and BDA can, indeed, (i) help predict communicable/non-communicable disease risk and prognosis, either at the individual and community/population levels (emergence/re-emergence and spread of an infectious disease outbreak, and non-communicable disorder surveillance, by means of dynamic risk-based early warning/monitoring systems), (ii) can enhance and strengthen laboratory functioning and capacity, in terms of identification of innovative biomarkers and precision diagnostics (clinical laboratory innovation) and infectious sample processing (public and global health laboratory innovation), (iii) predict treatment outcomes (individual health risk management) and monitor and forecast the effectiveness of the package of public and global health interventions implemented (community/population health risk management), and (iv) predict healthcare utilization (evidence-informed decision-making) (Table 1). Differently from conventional techniques, AI- and BDA-based technology enables the handling of highly heterogeneous data and the performance of dynamic models that are able to analyze new information and provide highly integrated, multi-level forecasts and predictions based on the processing of changing datasets. 

In the aftermath of the still ongoing COVID-19 pandemic, the future trend of AI and BDA in the healthcare field will be devoted to building a more healthy, resilient society, able to face several challenges arising from globally networked hyper-risks [40], including aging, multimorbidity, chronic disease accumulation, and climate change. 

All these challenges are highly interconnected. The latter, for instance, is expected, indeed, to profoundly disrupt human health, affecting respiratory and cardiovascular systems, as well as causing injuries and premature deaths [76] and increasing drug resistance [77]. AI- and BDA-enhanced precision clinical medicine can offer a viable solution to these problems. Furthermore, according to the “One Health” and “planetary health”/“clinical planetary health” approaches, human health should not be separated from the health of the ecosystem (humans, animals, plants, and the surrounding environment). Climate crisis seriously threatens the access to clean air, safe drinking water, and food, having an impact, especially on socially vulnerable communities, which are already marginalized, and have heightened vulnerabilities due to poverty, geographic location, sex and gender, Indigeneity, race, ethnicity, disability, sexual orientation and gender identity, job status, and workplace conditions. 

Socioeconomic inequalities and risks can exacerbate the effects of climate change itself, whereby sea-level rise and extreme weather events such as flash flooding and storm surges cause widespread devastation to coastal and inland communities across the globe. As urban areas expand, more people living and working near forested areas are likewise affected by greater exposure to disease vectors and longer average wildfire seasons, with catastrophic results [78].

AI- and BDA-based algorithms can help (i) identify climate-change tipping points by means of EWSs, (ii) strengthen the ability to detect such changes, (iii) devise mitigation responses and strategies, and (iv) guide and shape locally-informed policies. 

In conclusion, the novel use of AI and BDA is anticipated to uncover new links between climate, climate-related disaster exposure, and the burden of disease (especially, in terms of mental health) [78], helping policy- and decision-makers particularly in low- and middle-income countries (LMICs), such as those belonging to the Global South [79]. 

### 3.5. The Global South

The Global South is a grouping of countries and territories, from South America, Africa, and Asia, that are highly heterogeneous in terms of socioeconomic, cultural, and political characteristics, even though they share (i) obstacles and barriers to access to community and public healthcare services, (ii) the burden of disease generated by communicable disorders, (iii) food and (iv) job insecurity, and (v) the lack of state-of-art resources and infrastructure, necessary for their growth and development [80]. 

As such, the Global South differs from the concept of the “Hemispheric South”, which is a geographical one. The former is, instead, a meta-category or a broad umbrella term that comprises a vast array of decolonized nations, south of the old colonial centers of power. The health needs of the populations dwelling in the Global South are generally overlooked in the scholarly literature and are not prioritized in the political agenda [80]. 

As previously mentioned, AI and BDA can help pave the way for new opportunities both in the field of preventive and clinical medicine, advancing the design and implementation of personalized treatment and management, as well as precise clinical public and global health interventions. However, despite an accumulating body of evidence, only a few countries from the Global North are leading and shaping research and knowledge in the field, with findings that, as such, may not be generalizable and applicable/directly translatable to the Global South. To be really and truly meaningful, equitable, and impactful, there is an urgent need for socially and ethically responsible, inclusive, and collaborative/participatory AI and BDA, that can drive and support innovation in the Global South, in terms of research, training of qualified personnel, high-quality curated and integrated, diverse and representative, locally relevant and informed databases, algorithms, platforms, and infrastructure.

### 3.6. Ethical and Regulatory Challenges of Artificial Intelligence and Big Data Analytics for Clinical Public and Global Health in the Global South

“Responsible” AI and BDA can be defined as an array of innovative and emerging practices of intentionally devising, developing, implementing, and utilizing AI and BDA, to protect the public good and to empower the populations, especially vulnerable, at-risk communities, with a positive impact on society. Responsible AI and BDA are characterized by fairness, inclusiveness, transparency, accountability, explainability, human-centeredness, privacy, and security. “Explainable, trustworthy, responsible AI and BDA for social good” [81] can reduce and mitigate against some risks that can potentially arise when using AI and BDA for clinical public and global health, including (i) biases and lack of clarity for some AI and BDA algorithms, (ii) privacy issues for data utilized for AI model training, (iii) security issues and (iv) AI and BDA implementation-related responsibilities in real-world clinical public and global health settings.

These risks generated by some AI- and BDA-based technologies require a global governance infrastructure and a set of clear regulatory frameworks aimed at identifying high-risk AI and BDA applications, setting conformity assessments as well as requirements and obligations for AI- and BDA-based system developers, vendors, and users.

However, while the Global North is leading the debate on how to build, regulate, and make an “explainable, trustworthy, responsible use of AI and BDA for social good” [81], the Global South is significantly lagging behind in the efforts to devise an ethical governance architecture for AI and BDA [82,83,84]. In this regard, the Global South, an already excluded, marginalized, disenfranchised, oppressed, and discriminated reality, is exposed to further vulnerabilities, depending on “northern domination” [84], and having to “import digital technology, capital and modes of organization from these developed countries” [84].

**Figure 1:**
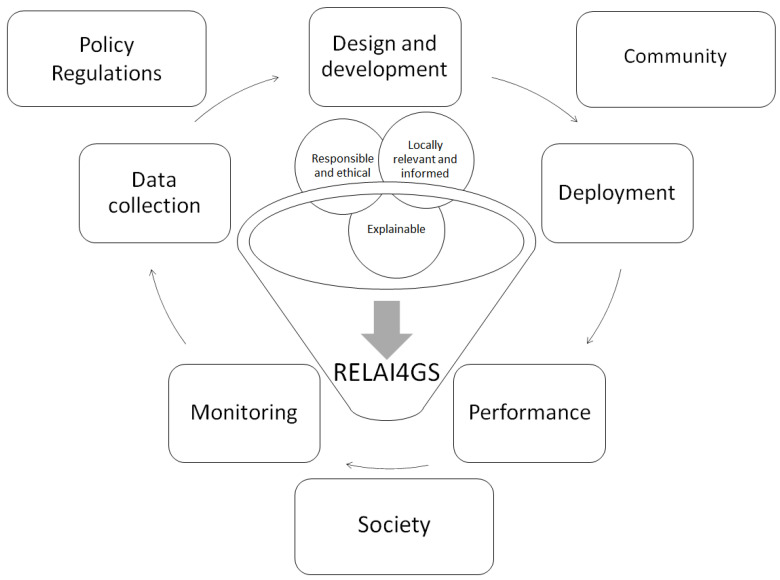
The framework adopted to address clinical public and global health needs in the Global South, leveraging and capitalizing on our experience with the “Africa-Canada Artificial Intelligence and Data Innovation Consortium” (ACADIC) Project in the Global South (“Responsible, Explainable, and Local Artificial Intelligence for Clinical Public and Global Health in the Global South”, REL-AI4GS). The diagram coherently shows the “how”, the “what”, and the “who” of our proposed framework. The inner shell (“how”) contains the set of ethical and legal rules and codes that should be designed in such a way that they are responsible (incorporating policy and regulations), locally relevant for communities, and explainable to society at large. Moreover, they should be applied and embedded all along the processes of AI solutions in the Global South. The medium shell (“what”) describes the processes that should be implemented in an iterative fashion (step 1: data collection, step 2: design and development, step 3: deployment, step 4: performance, and step 5: monitoring). The outer shell (“who”) contains all the relevant stakeholders and actors that should be involved.

Figure 1 and Table 3 depict the framework adopted to address clinical public and global health needs in the Global South, leveraging and capitalizing on our experience with the ACADIC Project in the Global South. We term this framework “Responsible, Explainable, and Local Artificial Intelligence for Clinical Public and Global Health in the Global South” (REL-AI4GS). The diagram coherently shows the “how”, the “what”, and the “who” of our proposed framework. The inner shell (“how”) contains the set of ethical and legal rules and codes that should be designed in such a way that they are responsible (incorporating policy and regulations), locally relevant for communities, and explainable to society at large. Moreover, they should be applied and embedded all along the processes of AI solutions in the Global South. The medium shell (“what”) describes the processes that should be implemented in an iterative fashion (step 1: locally relevant data collection, step 2: design and development of locally meaningful algorithms, step 3: deployment of locally relevant data, step 4: execution and performance of locally meaningful algorithms, and step 5: monitoring of the outcomes of the locally meaningful algorithms and identification (and removal) of potential biases). The outer shell (“who”) contains all the relevant stakeholders and actors that should be involved (Figure 1). 

### 3.7. Experience with the ACADIC Project in the Global South 

As previously mentioned, in the present paper, we will explore how AI and BDA can be exploited to address clinical public and global health needs in the Global South, based on our experience with the ACADIC Project in the Global South, focusing on the ethical and regulatory challenges we had to face, and leveraging the framework previously introduced (Figure 1, Table 3).

ACADIC has been devising and deploying AI- and BDA-based techniques to better understand the impacts of clinical public and global health interventions implemented during the COVID-19 pandemic in the Global South [85,86,87,88,89,90,91,92]. More specifically, ACADIC has been assisting policy- and decision-makers with the fight against COVID-19, including (i) monitoring and forecasting the growth and spread of COVID-19 at the local, state, and national levels [85,92], (ii) evaluating efforts to mitigate and control the spread [92], (iii) identifying trends in the disease infections, hospitalizations, and deaths [92], (iv) guiding purchase and allocation of health care resources [85], (v) guiding the collection of data (ensuring that data were disaggregated by race, gender, sexuality, class, geographic location, and Indigeneity) [92,93], (vi) guiding the implementation of vaccine roll-out and the development of effective, data-driven, evidence-informed immunization strategies, taking into account that available supply of vaccines in Africa was limited [87,90,91]; (vii) providing situational intelligence: on populations at risk, stage of the outbreak, the projected burden of illness, school/business/work closure and re-opening, etc. [88], (viii) nowcasting labor market flow [89], (ix) supporting race, gender, sexuality, class, geographic location, and Indigeneity, inclusive COVID-19 actions [92], (x) developing methodologies and technologies to describe contact mixing and transmission networks to quantify impacts of contact shifting and individual mobility [92], (xi) supporting transparent and responsible AI, data, and digital rights governance around COVID-19 and pandemic responses [92], (xii) strengthening data systems and information sharing about COVID-19, (xiii) building trust and combatting mis- and dis-information around COVID-19 [91], (xiv) optimizing public health system responses for patient diagnosis, care, and management [92], (xv) establishing sustainable collaborations among model developers, policymakers, community leaders, etc. [92], (xvi) preparing the next generation of leaders in infectious disease AI- and BDA-based modeling approaches in these countries [92], (xvii) working closely with public health agencies and other stakeholders to build trust and knowledge of AI-based models among key decision-makers [92], (xviii) developing stand-alone and predictive clinical public health decision support tools [92], and, (xix) creating a collaborative network that can respond rapidly to support decision-makers in each country to address infectious diseases or other disasters and emergency situations in general [92].

In South Africa, for example, ACADIC has been piloting the use of AI- and BDA-based modeling [94,95,96] to prioritize strategies in the COVID-19 vaccine roll-out in the Gauteng Province, using Deep Neural Network (DNN) algorithms [87]. In addition, ACADIC has been utilizing “non-conventional data streams” [75], such as Twitter [88,89], to better understand the “dynamics in sentiments toward community-based infectious diseases-related discussions” and to provide “city-level information to health policy in planning and decision-making regarding vaccine hesitancy” [91] and adopted macroeconomic responses to COVID-19 pandemic [89]. Subsequently, the ACADIC consortium has been transferring this pilot study to other African settings and contexts in terms of knowledge gained, lessons learned, and developed modeling techniques.

This has required accounting for country-specific differences in socio-demographic, epidemiological, and clinical variables, including rates of comorbidities. As such, ACADIC has been re-weighting the models initially developed for South Africa [86,90]. ACADIC has already carried this out with Botswana, and it is doing this for seven other African countries, in terms of validating and further correcting/adapting procedures in order to ensure that the assumptions of the model are aligned with the specific features of the selected country. 

Moreover, in terms of “explainable, trustworthy, responsible AI and BDA for social good” [81], ACADIC is also making efforts to expand our understanding of social disparities and vulnerabilities, which are of crucial importance in data gathering/collection, model design and implementation, and outcome interpretation [86]. A set of sources—such as detailed, locally informed geospatial maps—will be employed as inputs to our future models. Further, gender is a major variable impacting COVID-19, as well as other diseases and disease outbreaks, in terms of risk of developing communicable diseases, disease severity, response to treatment, adverse reactions to medications, and magnified social vulnerability [86]. For this reason, it is being actively incorporated and fully embedded by ACADIC across all AI- and BDA-based models, performing what is known as “Gendered Health Analysis” (GHA) [91]. 

ACADIC is also devising models that can assist clinical public and global health policy- and decision-makers develop optimal COVID-19 testing policies and mass vaccination strategies [86] as well as help in identifying some coronavirus variants of concern (VOCs) that may evade immunity conferred by vaccines and previous infections.

### 3.8. Specific Lessons Learned from the ACADIC Project in the Global South

Through the ACADIC project in the Global South, we have learned various lessons that will inform the execution of future programs and initiatives in the field of clinical public health and AI in the Global South. These lessons point to (Table 4):

#### 3.8.1. A Need for Partnerships with Community-Led Organizations (CLOs) and Community Healthcare Workers 

Partnering with CLOs and community healthcare workers helped us to acquire some of the data from communities and populations that do not visit the healthcare system. In our partnership with community health workers, we had them visit households in certain communities to collect data for us. Some of the CLOs that we partnered with already have networks set up in some of these communities and this made it very easy for us to acquire some of the “hard to get” data. For a meaningful change in the health of people in communities, it is important for solutions to be developed and scaled from the bottom up, as modeling and data collection must be community-focused, -owned, and -co-led [97]. 

#### 3.8.2. A Need for Buy-In from the Decision- and Policymakers

In most of the countries in the Global South, without “buy-in” from the government, it is difficult to influence policies or implement research results. For policy-driven research in the Global South, this is essential. Buy-in from the decision- and policymakers gave us access to data and the ability to influence the collection of data. Thanks to the fact that we had a seat at the table where decisions were made, we were able to influence data gathering in most of the countries and had it disaggregated/stratified by ethnicity, gender, sexuality, socio-economic class, geographic location, and Indigeneity to better understand how COVID-19 is disproportionately affecting socially vulnerable people. It enabled us to influence government communication strategies to address misinformation about the prevention and treatment of COVID-19.

#### 3.8.3. A Need for a Diverse Blend of Research and Implementation Experts

It is essential to put together a team with different expertise. The diverse expertise in our team enabled us to merge new data sources from science, technology, social, and cultural systems, in relation to opportunities and risks, centering local needs and knowledge while learning from all partners’ experiences. Being engaged with communities gave us the ability to adjust swiftly to changing circumstances.

#### 3.8.4. A Need for a Network to Create and Promote Mutual Support across the Network

For impactful research work in the Global South, it is important to create a network and have each team uniquely assemble its own strategy for meeting the program’s objectives and provide an opportunity for teams to learn from each other and an opportunity for knowledge transfer between the teams. In addition, the teams should be encouraged to draw on their own set of experiences, community partners, local context, constraints, and possibilities. By sharing their processes and results, the groups will draw out larger-scale comparisons, synergies, and conclusions in relation to the overall goal(s). It is important to provide an opportunity for them to ‘learn by doing and ‘in the community.’ Research that leads to meaningful change is new in most LMICs in the Global South given the history of colonialism and being “forced by circumstances” to rely on the formal colonial masters up to date for most of the innovations. Thus, it is necessary to hold each other’s hands and provide mutual support. The moment researchers become frustrated with research in most countries, if there is no one to provide them with the necessary support to keep going, they will give up. Learning from each other through weekly meetings, workshops, webinars, town hall meetings, etc., is very essential. We learned from other groups within the ACADIC consortium by having weekly meetings and regular workshops. The transnational partnership’s value-added for all partners in ACADIC is grounded in our sharing across contexts and specific situations regarding effective digital data generation, management, dissemination, and ways to address equity priorities for risk minimization by amplifying the voices and agency of marginalized and highly impacted communities.

#### 3.8.5. A Need for Novel Data-Gathering Techniques, Including Citizen Science/Participatory Science and Simple, Anonymous Digital Platforms for Data Reporting

It is imperative to harness data from all available sources including unconventional sources to make up for the gaps that exist in data. We have used unconventional datasets that include, household visits by community health workers, voice scripts, chatbots, Twitter, Google searches, community-level Reddit, WhatsApp, Facebook, etc., to inform health needs about COVID-19 [88,89,91]. In addition, there is a need for novel data-sharing and dissemination to marginalized groups, not just governmental agencies, for incorporation into informal-sector and community-based initiatives.

#### 3.8.6. A Need for AI and Big Data Governance and Legislation for the Global South

We developed a framework that takes into account regulatory and ethical aspects (policies, regulations), as well as community and societal needs. We extensively searched the literature (either scholarly/peer-reviewed) [98] and we identified 35 adjectives that were mapped to three dimensions (dimension R: responsible, dimension E: explainable, and dimension L: local). The processes of (i) data collection, (ii) design and development, (iii) deployment, (iv) performance and (v) monitoring are implemented iteratively and progressively refined until each potential gap is filled in. A set of iniquity/disparity metrics is adopted to track risks of biases and vulnerabilities, that need to be addressed. 

#### 3.8.7. A Need for Strengthening AI- and BDA-Related Funding in the Global South

Science granting councils and local funding agencies in the Global South suffer from several, interrelated shortcomings and challenges, including a lack of adequate capacity, coordination, and implementation of research funding policies. Local policy frameworks and enabling structures should be developed and/or strengthened to better support the local communities of scholars and researchers. Moreover, public-private sector partnerships should be incentivized.

#### 3.8.8. A Need for Strengthening AI- and BDA-Based Modeling Capacity in the Global South

AI- and BDA-based modeling has attracted increasing interest from policy and decision-makers across countries in the Global South, including Africa. AI- and BDA-based modeling can, indeed, help them shape their local, regional, and national Strategic Plans in an informed, evidence-based, and data-driven fashion, tracking their progress and monitoring their effectiveness. 

However, there still exists a significant knowledge power imbalance between a few countries leading in AI- and BDA-based modeling and the rest of the world. As such, the AI modeling ecosystem and the research and development (R&D) landscape across the countries in the Global South should be strengthened, in terms of adequate qualified personnel of researchers and innovators, as well as infrastructure and algorithms/tools. 

## 4. Discussion

In the present paper, we explored how AI and BDA can help address clinical public and global health needs in the Global South, leveraging and capitalizing on our experience with the ACADIC Project in the Global South, and focusing on the ethical and regulatory challenges we had to face.

Whilst clinical public health is at the intersection of clinical medicine and public health, clinical global health is the practice of clinical public health in the Global South. As such, clinical public and global health represent vital approaches, instrumental in combining a community/population perspective with clinical practice, identifying health needs, systematically addressing the determinants of health, better coordinating and integrating the delivery of healthcare provisions, reaching the goals of population’s health and well-being, strengthening health promotion, health protection, and health equity, and closing disparities and gaps. Clinical public and global health are called to respond to the more pressing healthcare needs and challenges of our contemporary society, for which AI and BDA can help unlock new options and perspectives. In the aftermath of the still ongoing COVID-19 pandemic, the future trend of AI and BDA in the healthcare field will be devoted to building a more healthy, resilient society, able to face several challenges arising from globally networked hyper-risks, including aging, multimorbidity, chronic disease accumulation, and climate change.

Based on the existing literature and our experience, we identified the following lessons (i) strengthening local research and healthcare capacity in the Global South, (ii) strengthening local epidemic/pandemic management planning, including the establishment and development of networks with simulator users in the Global South, (iii) a need for locally informed models in the Global South, (iv) a need for flexible modeling frameworks to respond rapidly to future emergencies in the Global South, (v) limitations and shortcomings of modeling should be communicated clearly and consistently to end users in the Global South, (vi) systematically monitoring the use and implementation of models in the process of decision-making in the Global South, (vii) a need for strengthening AI- and Big Data-related funding in the Global South, and (viii) a need for strengthening AI- and BDA-modeling capacity in the Global South.

This is in line with other experiences of authors from the Global South, which reaffirm that equity in health is paramount, being “the core value of health for all” [99], as advocated by the Alma-Ata declaration of 1978. This goal is even more ambitious in a world and society where inequities are prevailing and poverty is increasingly widening, with the emergence/re-emergence of new illnesses [100] and the accumulation of chronic diseases [101], which put a significant strain on already weakened healthcare systems [99]. 

As such, carrying out health research is vital, even though, in some resource-limited areas and environments, it may be particularly complex and difficult, with a limited capacity to undertake and translate research into practice [102]. There, it is fundamental to strengthen and develop sustainable health research capacity in the Global South [102]. Key enablers were identified in (i) ensuring adequate, vigorous funding, (ii) building effective stewardship and establishing equitable and sustainable research collaborations and partnerships, (iii) mentoring and training next-generation researchers, and (iv) effectively linking research-related outcomes to policies and practices [102].

Researchers and scholars from the Global South are terribly under-represented in research [103,104,105,106,107,108,109,110], project leadership and management, authorship, and funding allocations [106], paradoxically even in research concerning the Global South itself [103,104]. Therefore, decolonizing global health research, partnerships, and outreach could mean counteract and mitigate against global health iniquities [105,106,107,108,109]. This “paradigm shift” [106] implies (i) redefining equitable and fair models and best practices of collaboration and (ii) implementing mechanisms to monitor and track progress [106], as well as (iii) recognizing “non-western forms of knowledge and authority”, acknowledging discrimination and disrupting “colonial structures and legacies that influence access to healthcare” [108].

## 5. Limitations

The present study has some limitations, that should be acknowledged. The literature review was narrative rather than systematic and the qualitative part of the study was preliminary. As such, further research is needed. Moreover, only a limited number of countries from the Global South could be included and the findings of the current review may not be generalizable to other realities and contexts from the Global South. 

## 6. Conclusions

Through a narrative literature review and our experience with the ACADIC project, we have learned many lessons that will inform the execution of future initiatives in the Global South. If all these lessons are properly addressed, it will be possible to carry out responsible, inclusive, and impactful AI- and BDA-based research in the healthcare sector that will provide global benefits However, our literature review is narrative and our qualitative analysis preliminary. As such, further research in the field is warranted. More in detail, future studies should conduct high-quality systematic reviews on some (sub-)topics such as clinical public and global health, as well as on digital health in the Global South. Moreover, new interactive, web-based trackers and indicators, such as the “Global Digital Health Index” (GDHI), should be developed and validated to evaluate and monitor the progress toward inclusive, equitable, responsible, and locally championed AI and digital health throughout the world, with a focus on the Global South. 

## Figures and Tables

**Table 1:** The four major components of clinical medicine, and public and global health.

Component	Definition
Prevention	Disease and disease outbreak monitoring/surveillance and early warning systems
Identification	Clinical, public, and global health laboratory innovation
Risk management	Disease and disease outbreak risk reduction and mitigation
Decision-making processes	Evidence-informed and data-driven

**Table 2:** Ways of approaching health and well-being at the individual and community/population levels.

Discipline/Specialization	Definition	References
Clinical medicine	Aimed at treating “sick individuals”	[4,5]
Public health	Aimed at treating “sick populations” by preventing communicable and noncommunicable diseases, counteracting/mitigating against their burden, prolonging life, preserving the quality of life, and promoting health and well-being	[14,15]
Clinical public health	Combining clinical medicine and public health, integrating primary care, clinical practice, disease management and treatment, and prevention	[11]
Global health	Practicing public health with a focus on resource-limited settings and contexts, by improving health and well-being and achieving health equity for all populations worldwide	[21,22]
Clinical global health	Practicing clinical public health with a special focus on health issue management in resource-limited settings and contexts	[17,18]

**Table 3:** The three components of the “Responsible, Explainable, and Local Artificial Intelligence for Clinical Public and Global Health in the Global South” (REL-AI4GS) framework.

Component	Definition
Responsible	Accountable, auditable, compliant, ethical, respectful, safe, secure
Explainable	Equitable, fair, impactful, interpretable, meaningful, reliable, reproducible, transparent, trustworthy, unbiased
Local	Autonomous, caring, connecting, decolonized, human- and community-centered, inclusive, intentional, intersectional, just, participatory, practical, protecting, process-based, sustainable

**Table 4:** The results of the qualitative analysis, which has enabled the identification of the lessons learned from the ACADIC Project in the Global South.

Lesson	Definition
Lesson n. 1	A need for partnerships with community-led organizations (CLOs) and community healthcare workers
Lesson n. 2	A need for buy-in from the decision- and policymakers
Lesson n. 3	A need for a diverse blend of research and implementation experts
Lesson n. 4	A need for a network to create and promote mutual support across the network
Lesson n. 5	A need for novel data-gathering techniques, including citizen science/participatory science and simple, anonymous digital platforms for data reporting
Lesson n. 6	A need for AI and Big Data Governance and Legislation for the Global South
Lesson n. 7	A need for strengthening AI- and BDA-related funding in the Global South
Lesson n. 8	A need for strengthening AI- and BDA-based modeling capacity in the Global South

## Data Availability

All data are within the paper.
